# A needs assessment of palliative care education in neurology residency programs in Brazil

**DOI:** 10.1055/s-0045-1811232

**Published:** 2025-09-08

**Authors:** Maiara Silva Tramonte, Ana Claudia Pires Carvalho, Mariana Soares Pinheiro, Camila Galvão Lopes, Luciana Oliveira Neves, Laura Cardia Gomes Lopes

**Affiliations:** 1 Universidade Estadual Paulista “Júlio de Mesquita Filho”, Faculdade de Medicina de Botucatu, Departamento de Neurociências e Saúde Mental, Botucatu SP, Brazil.; 2 Universidade de São Paulo, Faculdade de Medicina, Departamento de Neurologia, São Paulo SP, Brazil.; 3 Universidade de Fortaleza, Centro de Ciências da Saúde, Departamento de Saúde Pública, Fortaleza CE, Brazil.

**Keywords:** Education, Medical, Internship and Residency, Neurology, Palliative Care

## Abstract

**Background:**

The importance of integrating palliative care (PC) competencies into the management of neurological diseases is increasingly acknowledged. The National Medical Residency Program Commission (NMRPC) mandates the inclusion of PC in the curricula of neurology residency programs (NRPs).

**Objective:**

To evaluate the implementation of PC training in NRPs across Brazil and to identify barriers to its effective integration.

**Methods:**

We conducted a cross-sectional study using an anonymous online survey distributed to all NRP directors in Brazil through the Brazilian Academy of Neurology (BAN). The survey, approved by the institutional ethics committee, comprised 19 questions assessing PC training in NRPs. The responses were analyzed descriptively using IBM SPSS Statistics for Windows (IBM Corp.) software, version 26.0.

**Results:**

The survey achieved a 32% (29 programs) response rate. Of the respondents, 82.8% (24 programs) reported the presence of an institutional PC team, and 75.9% (22 programs) incorporated PC training into their programs. However, only 1 neurology faculty member had formal PC training, and 37% of the directors expressed dissatisfaction with the quality of PC education. Key barriers included limited faculty availability (72.4%), lack of PC expertise (69%), and insufficient teaching time (58.6%).

**Conclusion:**

Palliative care training in Brazilian NRPs lacks consistency and systematic implementation. There is an urgent need for enhanced faculty training and strategic interventions to address structural and curricular barriers to improve PC education.

## INTRODUCTION

Neurological disorders are increasingly prevalent and represent the second leading cause of death globally. 1 Many of these conditions result in significant physical or cognitive impairments, profoundly impacting patients' quality of life. These challenges require a range of competencies, including palliative care (PC), throughout the disease trajectory. 2 3

Palliative care is an approach recommended for patients living with life-threatening illnesses, from diagnosis to family bereavement support. It focuses on clear communication, conflict resolution, patient-centered care, relief of physical and psychological symptoms, and addressing spiritual distress. The aim is to improve the quality of life, outline advance care directives, and facilitate a peaceful death. 4

Neurologists play a pivotal role in managing neurological conditions not only in the diagnosis and management in the initial stages, but also throughout incurable neurological conditions, estimating the prognosis, establishing goals of care, integrating the interdisciplinary team, and evaluating any acute intercurrences that they may arise over time. 3 Thus, integrating PC principles across all stages of neurological diseases is essential, not solely in their terminal stages. 5 6

Palliative care is categorized into primary care, delivered by healthcare professionals such as neurologists who initiate care for serious illnesses, and specialized care, provided by PC specialists. 5 7 Strategic priorities to advance PC in neurology include enhancing PC training for neurologists and improving neuropalliative care education for general PC specialists. 5

The significance of PC in neurological diseases has grown, which is evidenced by an increase in educational initiatives, guidelines, and publications. 2 8 9 However, currently there is no guidance on how to teach PC to neurologists, nor is there a standardized assessment of whether this knowledge was taught properly. 10 11

The provision of PC education to neurologists is not only essential; it is also mandatory in Brazil. 12 For over two decades, national and international organizations, such as the American Academy of Neurology (AAN) and the Accreditation Council for Graduate Medical Education (ACGME), have emphasized the importance of foundational training in palliative care. 9 However, the development of standardized educational resources, curricula, and competencies remains limited, and a notable challenge is the scarcity of neurology programs with specialized professionals in neuropalliative care. 10 11 13 14

Previous research, such as a United States (US) survey by Creutzfeldt et al., 15 revealed that 53% of neurology residents received no PC training, with an average knowledge score of 44%. Another US study found that 20% of programs offered no PC education, and 42% of directors were dissatisfied, citing barriers such as limited time, untrained faculty, and lack of expertise. 8

The present study aims to evaluate the current state of PC education in Brazilian neurology residency programs (NRPs) and identify key challenges to its integration, contributing to the development of targeted strategies for improvement.

## METHODS

### Design

The current cross-sectional study used an anonymous online survey distributed via email to all NRP directors in Brazil, facilitated by the Brazilian Academy of Neurology (BAN). The study was approved by the institutional ethics committee (CAAE: 51867221.6.0000.5411). The participants provided informed consent online, and participation was voluntary, without financial incentives.

### Survey development

The survey comprised 16 multiple-choice and 3 Likert-scale questions ( **Supplementary Material** , available at https://www.arquivosdeneuropsiquiatria.org/wp-content/uploads/2025/06/ANP-2025.0035-Supplementary-Material.docx ), adapted from a previous US study, 8 with modifications to reflect the Brazilian context. The questions addressed PC workload, training modalities, theoretical study requirements, directors' perceptions of residents' PC mastery, and the importance they attach to teaching PC to neurology residents.

### Survey distribution

All 90 NRPs listed by the BAN were contacted 3 times at monthly intervals between March and May 2022. The survey was hosted on the REDCap platform (Vanderbilt University), with invitations, informed consent forms, and study details sent via BAN's official email to enhance credibility and avoid spam filters. The survey targeted NRP directors, assistant directors, or supervising preceptors most familiar with the curriculum.

### Data analysis

Data were exported from REDCap to the IBM SPSS Statistics for Windows (IBM Corp.) software, version 26.0, for the descriptive analysis, with the data expressed as mean and standard deviation values or median and interquartile range (IQR) values. Due to the small sample size, no subgroup comparisons were performed.

## RESULTS

The survey achieved a 32% response rate (29 out of 90 programs). The descriptive data are presented in Table 1 .

**Table 1:** Baseline characteristics of the residents in Brazil's adult neurology residency programs

N = 29 (response rate: 32%)		Median (25th percentile–75th percentile)/n (%)
Region	South	9 (31.03)
	Southeast	14 (48.27)
	Midwest	–
	North	2 (6.89)
	Northeast	4 (13.79)
Number of residents per year		4 (3–6)
Number of neurology faculties		12 (10–15)
Role of the person who answered the survey	Program director	15 (51.72)
	Resident's preceptor	12 (41.37)
	Assistant program director	2 (6.89)
Type of service	University with a public teaching hospital	23 (79.31)
	University with a private teaching hospital	1 (3.44)
	College with a public hospital	1 (3.44)
	College with a private hospital	0 (0)
	Other*	4 (13.79)
Is palliative care available in your service?	Yes	24 (82.75)
	No	5 (17.24)
Who teaches palliative care?	Neurology faculty without formal training in palliative care	12 (41.37)
	Faculty of palliative care	20 (68.96)
	Other faculty (not palliative care or neurology)	1 (3.44)
	Neurology faculty with formal training in palliative care**	4 (13.79)
	The chaplains	0 (0)
	Does not know	0 (0)
	Other	0 (0)
	Does not apply	1 (3.44)
Is there palliative care teaching to the resident?	Yes	22 (75.86)
How satisfied are you with the teaching of palliative care at your institution?	Extremely satisfied	2 (6.89)
	A little satisfied	16 (55.17)
	A little dissatisfied	4 (13.79)
	Extremely dissatisfied	7 (24.13)
Do you use any educational materials to teach the resident palliative care?	Yes	12 (41.37)
	Articles	5 (17.24)
	Speeches	1 (3.44)
	Brazilian National Academy of Palliative Care Manual	2 (6.89)
	Classes	3 (10.34)
	International guidelines	1 (3.44)
	Material from the Brazilian Ministry of Health	1 (3.44)

Notes: *Private hospital without university affiliation, philanthropic hospital accredited by the Brazilian Unified Health System (Sistema Único de Saúde – SUS, in Portuguese), and university-affiliated teaching hospital. **They had only 1 member with palliative care training per program

**Figure 1:**
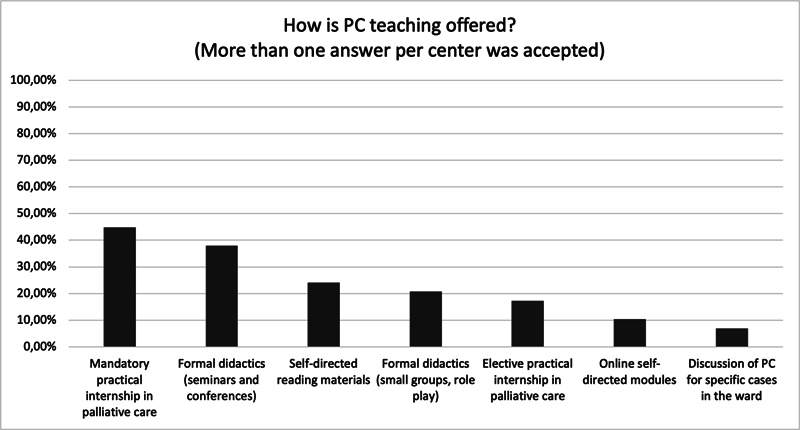
Methods of providing PC education in NRPs (multiple responses per center allowed).

**Figure 2:**
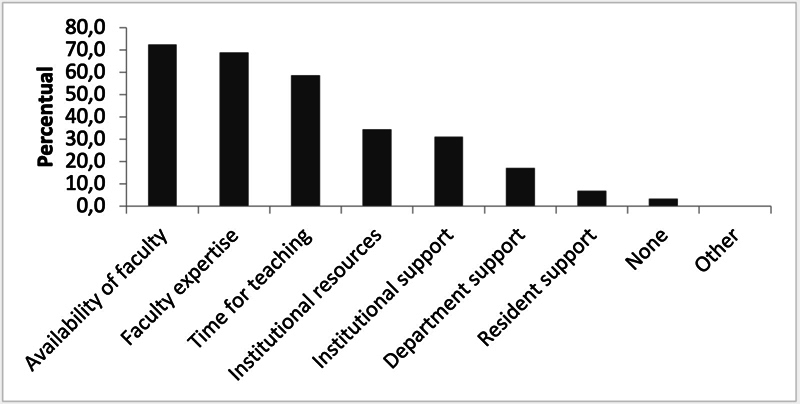
Main barriers to the implementation of palliative care (PC) education in neurology residency programs (NRPs) (multiple responses per center allowed).

Of the respondents, 82.8% (24 programs) reported an institutional PC team, and 75.9% (22 programs) included PC training. Only 4 neurology faculty members across all programs had formal PC training. Figure 1 shows how PC education is conducted in the institutions. Additionally, 37% of NRP directors expressed dissatisfaction with the quality of PC education. The key barriers to PC education included limited faculty availability (72.4%), lack of PC expertise (69%), and insufficient teaching time (58.6%), as shown in Figure 2 .

Three Likert-scale questions assessed the directors' perceptions of PC teaching topics: the importance they assign to teaching specific topics, their comfort in providing PC, and residents' training levels. The greatest discrepancies between importance and comfort were observed in withdrawing or initiating life-prolonging therapies and addressing ethical and legal aspects.

**Figure 3:**
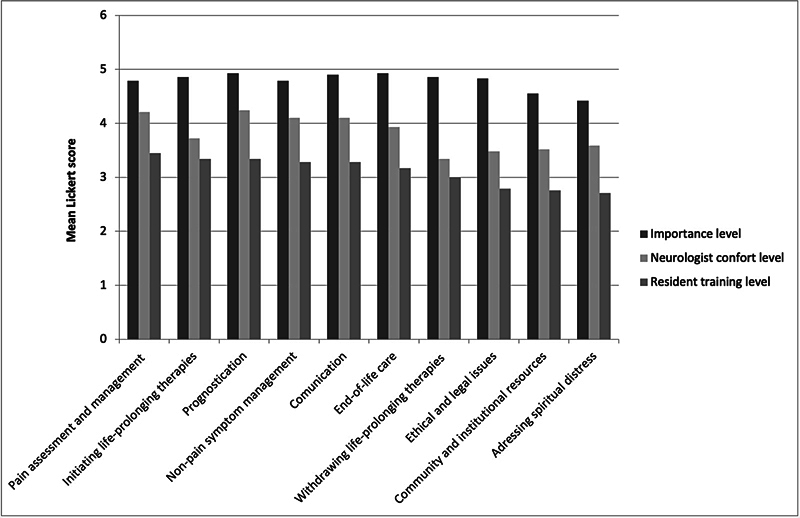
Comparison of importance and comfort levels among NRP directors and resident training levels on key topics.

Addressing spiritual distress was deemed least important and the area of lowest comfort. The topics least covered in training included ethical and legal aspects, use of community/institutional resources, and spiritual care ( Figure 3 ).

Table 2 illustrates the gap between the importance assigned to PC topics (all scoring > 4 on the Likert scale) and the directors' comfort levels (scores of 3–4). All topics remained significant after the application of Bonferroni correction.

**Table 2:** Neurology residency program directors' ratings: importance and comfort level with key topics

	Importance score assigned to the topic (1–5 Likert 1-5 scale)		Comfort level with the topic (1–5 Likert scale)		
	Mean		Mean		*p* ^β^
Pain assessment and management	4.79 ± 0.491		4.21 ± 0.902		< 0.0001**
Initiating life-prolonging therapies	4.86 ± 0.351		3.72 ± 1.131		< 0.0001**
Prognostication	4.93 ± 0.258		4.24 ± 0.739		< 0.0001**
Non-pain symptom management	4.79 ± 0.491		4.1 ± 0.724		< 0.0001**
Communication	4.9 ± 0.31		4.1 ± 0.939		< 0.0001**
End-of-life care	4.93 ± 0.258		3.93 ± 0.998		< 0.0001**
Withdrawing life-prolonging therapies	4.86 ± 0.351		3.34 ± 1.078		< 0.0001**
Ethical and legal issues	4.83 ± 0.384		3.48 ± 1.153		< 0.0001**
Community and institutional resources	4.55 ± 0.572		3.52 ± 1.184		< 0.0001**
Addressing spiritual distress	4.42 ± 0.688		3.59 ± 0.983		< 0.0001**

Notes: **Statistically significant after the application of Bonferroni correction; Mann-Whitney test for comparison between two independent groups.

## DISCUSSION

The current survey revealed a lack of standardized PC training in Brazilian NRPs. In our sample, 75.9% of the responding programs reported providing some PC education for neurology residents. However, the content and format of instruction varied widely: some programs provided theoretical instruction through seminars, while others offered practical training without specifically addressing the unique aspects of neurological cases. This variability mirrors global challenges in integrating structured PC education into medical training. 8

It is crucial to recognize that primary PC care should be integrated into the management of patients with neurological diseases, given their high symptom burden and impact on quality of life. However, the lack of specific training in neuropalliative care represents a significant barrier to delivering appropriate care. In this survey, only 1 neurology faculty member across all responding programs had formal training in PC. The predominance of non-neurologist PC educators and oncology-focused PC services may further hinder tailored care for neurological patients. Therefore, promoting specialized PC training for neurologists is essential to improve the quality of care for this patient population. 8 16

Barriers to PC training in NRPs, including limited faculty, institutional resistance, scarce resources, and weak neurology–PC integration, align with global findings. 14 In Brazil, the directors identified faculty availability (72.4%), lack of expertise (69%), and insufficient teaching time (58.6%) as primary challenges, reflecting systemic issues requiring institutional investment in faculty development and interdisciplinary collaboration.

The results reveal a notable discrepancy between the importance assigned by NRP directors to PC topics and their reported comfort level in addressing these subjects. As shown in Table 2 , while all 10 topics were rated as *highly important* (with scores exceeding 4 on the Likert scale), the directors' comfort levels with these subjects were consistently lower, ranging from 3 to 4. This disparity suggests a significant gap in preparedness and confidence among directors to engage with these critical areas of PC. Similarly, in a recent Italian study 17 involving neurology residents, the majority reported recognizing the relevance of PC and advanced care planning (ACP), but only a small proportion had received formal education or practical exposure to these topics during residency. The significance of all topics in the present study, even after applying Bonferroni correction for multiple comparisons, underscores the consistency and robustness of these findings and reinforces the need for targeted educational interventions to enhance directors' proficiency and improve the quality of PC training in NRPs.

The results of the survey also indicate that there is a significant discrepancy between the perceived importance of certain PC-related topics among NPR directors and the actual level of training their residents receive, suggesting a gap between ideal and actual teaching practices. The most pronounced gaps were observed in the areas of withdrawal of life-prolonging therapies, ethical and legal aspects, and the initiation of life-sustaining interventions. This finding is consistent with those of previous studies 5 8 13 that highlight the limited integration of PC into neurology curricula, particularly regarding complex decision-making at the end of life. Kluger et al. 13 (2022), for example, noted that, although neurologists often face ethically challenging decisions, formal training on such topics remains inconsistent and undervalued across many programs. Moreover, Mehta et al. 8 (2018) found that neurology program directors in the US similarly recognized the importance of CP but reported limited implementation due to structural and curricular barriers. These results reinforce the need to prioritize structured education in ethical and legal dimensions of care, especially in situations involving the withholding or withdrawal of life-sustaining treatment, scenarios frequently encountered but rarely addressed in formal training.

Addressing spiritual distress was least prioritized and least comfortable for directors, suggesting a need for broader training in holistic care, as psychosocial and spiritual domains are often underrepresented in neurology education. Bombaci et al. 17 (2024) also noted a tendency for PC education to emphasize physical symptom management while underrepresenting psychosocial and spiritual domains. Comprehensive PC requires addressing psychological, social, and spiritual needs alongside physical symptoms. Efforts to enhance residents' and directors' training in these areas are essential to align with PC's holistic philosophy.

It is important to recognize that human suffering is not limited to the physical sphere, and that addressing the psychological, social, and spiritual needs of patients and families is essential to provide comprehensive PC. 6 18 Therefore, efforts should be made to improve the training of neurology residents in these areas, as well as to raise awareness among NRP directors of the importance of addressing these aspects of care in the training of future neurologists.

Palliative care education should begin in undergraduate medical training. In Canada, a 2018 survey 18 found that only 11% of medical schools included a mandatory PC internship. Similarly, a Brazilian study 10 conducted in the same year but published in 2021 revealed that only 44 medical schools (14%) incorporated PC in their curriculum, and the content was predominantly theoretical. Although PC has recently been recognized as a public health policy in Brazil, 19 its implementation remains incomplete. Significant regional disparities persist in access to both healthcare and education. It was only in 2023 that PC was formally integrated into the national medical curriculum, following the approval of the respective resolution at the end of 2022. 10 20

The provision of PC education to neurologists is not only essential; it is also mandated in Brazil. 12 However, the implementation of this training faces numerous barriers. To overcome these challenges, it is crucial to identify the specific needs of individual institutions, promote the training of additional specialists, establish collaborative partnerships, and develop curricula that address the unique needs of neurological patients. Meanwhile, remote training programs focused on the discussion of real-world cases may be as an effective strategy, acknowledging that comprehensive education and training require substantial time and institutional investment. 13 21 22

The current study had a low response rate, of 32%, which is common in online survey research and consistent with other international studies on similar topics. 8 17 The sample may be biased, since only a minority of centers responded, and these may be the ones most interested in the topic. Additionally, the highest response rate comes from the Southeastern and Southern regions of Brazil, which are known to be more developed regions and to have more PC services according to the Brazilian National Academy of Palliative Care (Academia Nacional de Cuidados Paliativos, ANCP, in Portuguese). 16 Moreover, most responses were from public universities with teaching hospitals that have more PC services and policies. Therefore, the extent of the problem may be underestimated.

It is noteworthy that the similarities between the Brazilian (the present) and American 8 studies suggest that these barriers to PC training in NRPs are not exclusive to one country. Despite the limitations of both studies (32% in the Brazilian study and 35% in the American study 8 ), they offer valuable insights into the current state of PC education in NRPs and suggest areas for improvement. Further research and interventions are needed to overcome these barriers and enhance the integration of PC into neurology training globally.

In conclusion, the current study reveals that PC education in Brazilian NRPs is inconsistently implemented, with significant variability in teaching methods and content. The key barriers include a lack of trained faculty, limited expertise, and insufficient teaching time. These findings provide critical insights for the development of targeted strategies and policies to standardize and enhance PC education in NRPs, ultimately improving care for patients with serious neurological conditions.
